# Application of retaining the vessels around the orbicularis oculi muscle in blepharoplasty

**DOI:** 10.1111/jocd.16400

**Published:** 2024-05-20

**Authors:** Dan Li, Han Yan, Hengshu Zhang, E Yang, Xinhao Liu

**Affiliations:** ^1^ Department of Plastic and Burn Surgery The First Affiliated Hospital of Chongqing Medical University Chongqing China

**Keywords:** blepharoplasty, patient satisfaction, postoperative swelling, vessel retention

## Abstract

**Objective:**

To investigate the effect of retaining the vessels around the orbicularis oculi muscle on reducing local swelling after blepharoplasty.

**Methods:**

A total of 309 patients undergoing blepharoplasty (total incision) were observed and randomly assigned to three groups; (A) conventional operation; (B) preservation of deep vessels; (C) preservation of anterior vessels of orbicularis oculi muscle. The groups were compared based on intraoperative blood loss, operation time, swelling, satisfaction, and complications.

**Results:**

Among the 309 patients, 39 were lost to follow‐up. c Additionally, A had the shortest operation time, followed by C with slightly longer duration. On the 7th day, 15th day, and 1 month after surgery, both B and C demonstrated significantly lower levels of swelling compared to A. Moreover, patient satisfaction was higher in B and C than in A.

**Conclusion:**

Retaining either superficial or deep veins of the orbicularis oculi muscle can effectively reduce short‐term postoperative swelling. However, when retaining the superficial central group of this muscle during surgery, it is crucial to strictly control the amount of surrounding tissue around vessels.

## 1 INTRODUCTION

Postoperative swelling is one of the most common stress responses to trauma,^1^ and is currently one of the most concerned issues for patients. No matter what kind of surgery, some arteries and veins, lymphatic vessels, and nerves will be cut off, causing venous and lymphatic reflux blockage and local sensory disturbance, which need to be reestablished after surgery. Taylor GI^2^ showed that capillaries can germinate at least 5 days after injury, and mature blood vessels form 15 days later. In the process of postoperative repair, local blood reflux blockage is one of the causes of postoperative swelling of eyelids before new capillaries germinate after injury. For blepharoplasty, minimally invasive techniques can minimize damage to the vessels and lymphatic vessels of the upper eyelid.^3^, ^4^ However, this approach may not be suitable for all individuals. It is important to note that many Asians have excessive orbital septum fat or ROOF layer fat, which can result in puffiness.^5^, ^6^ Moreover, a significant number of patients require partial removal of upper eyelid skin for optimal outcomes, a challenge that cannot be effectively addressed by minimally invasive methods. The total incision method remains the prevailing choice for double eyelid surgery currently. However, as society progresses, an increasing number of individuals seeking beauty are pursuing optimal aesthetic outcomes, minimal postoperative swelling, and swift recovery for social and work engagements. Various scholars have conducted diverse investigations into reducing postoperative swelling with successful outcomes. This study aims to explore the impact of preserving deep and superficial vessels of the orbicularis oculi muscle on the extent of swelling during blepharoplasty.

## 2 MATERIALS AND METHODS

### 2.1 Patients

A total of 309 patients who underwent upper eyelid surgery between January 2021 and October 2022 were observed, comprising 265 females and 44 males within the age range of 18–45 years. Inclusion criteria: (1) Patients who are planning to undergo primary blepharoplasty using the total incision technique, and willing to participate in the follow‐up process. Exclusion criteria: (1) Previous history of upper eyelid; (2) Concurrent ptosis; (3) Prior periocular trauma; (4) History of chronic periocular diseases; (5) Systemic conditions such as uncontrolled diabetes, unexplained fever, severe cardiovascular disease, nephrotic syndrome, coagulation dysfunction, autoimmune disorders, etc.

The surgical procedures were performed by the same orthopedic surgeon, and all patients received identical postoperative care. This study was conducted in accordance with the principles outlined in the Helsinki Declaration and obtained approval from the Ethics Committee.

### 2.2 Grouping and surgical process

All patients were allocated into three groups using the random number table method, with 103 patients in each group. (A) Conventional—complete excision of the anterior orbicularis oculi vein network, along with removal of a major portion of the anterior tarsus vein network (deep venous plexus of the orbicularis oculi). (B) Deep vessel preservation—complete excision of the anterior orbicularis oculi vein while preserving the deep anterior tarsus vein network. (C) Anterior orbicularis oculi vessel preservation—selective preservation of a subset of anterior orbicularis oculi veins (typically coarser ones located centrally), without deliberate preservation of most deep anterior tarsus veins.

#### 2.2.1 Conventional surgery

The patient is seated, and the width and shape of the double eyelid line are customized based on the patient's preferences. The width ranges from 5.8 to 7 mm, while the length is approximately 3 cm. The extent of skin removal depends on the degree of relaxation, varying between 1.5 and 3.4 mm. Local anesthesia is administered using a mixture of 2% lidocaine and 1/200000 epinephrine. The full layer of skin was incised along the incision marker line with a No.11 blade, and the excess skin, orbicularis oculi muscle, and pre‐meibomian fascia, and intraorbital fat and ROOF fat were removed. During the operation, all the anterior vessels of the orbicularis oculi muscle below the incision were severed, and most of the anterior vessels of the tarsus were not intentionally preserved. After ensuring adequate hemostasis, sutures made with a fine nylon thread (7–0) are employed to close up the incisions by passing through layers including lower lip skin, lower lip orbicularis oculi muscle, near levator aponeurosis upper edge of tarsus, upper lip orbicularis oculi muscle, and finally upper lip skin sequentially.

#### 2.2.2 Deep vessel preservation

The design and local anesthesia techniques remained unchanged. The skin and orbicularis oculi muscle were trimmed as necessary, completely severing the venous network in front of the muscle while preserving the vascular network in front of and perpendicular to the tarsus (Figure 1A). Excess intraorbital septum fat and ROOF layer fat were excised, followed by thorough hemostasis prior to suturing and fixation using the same method as before.

**FIGURE 1:**
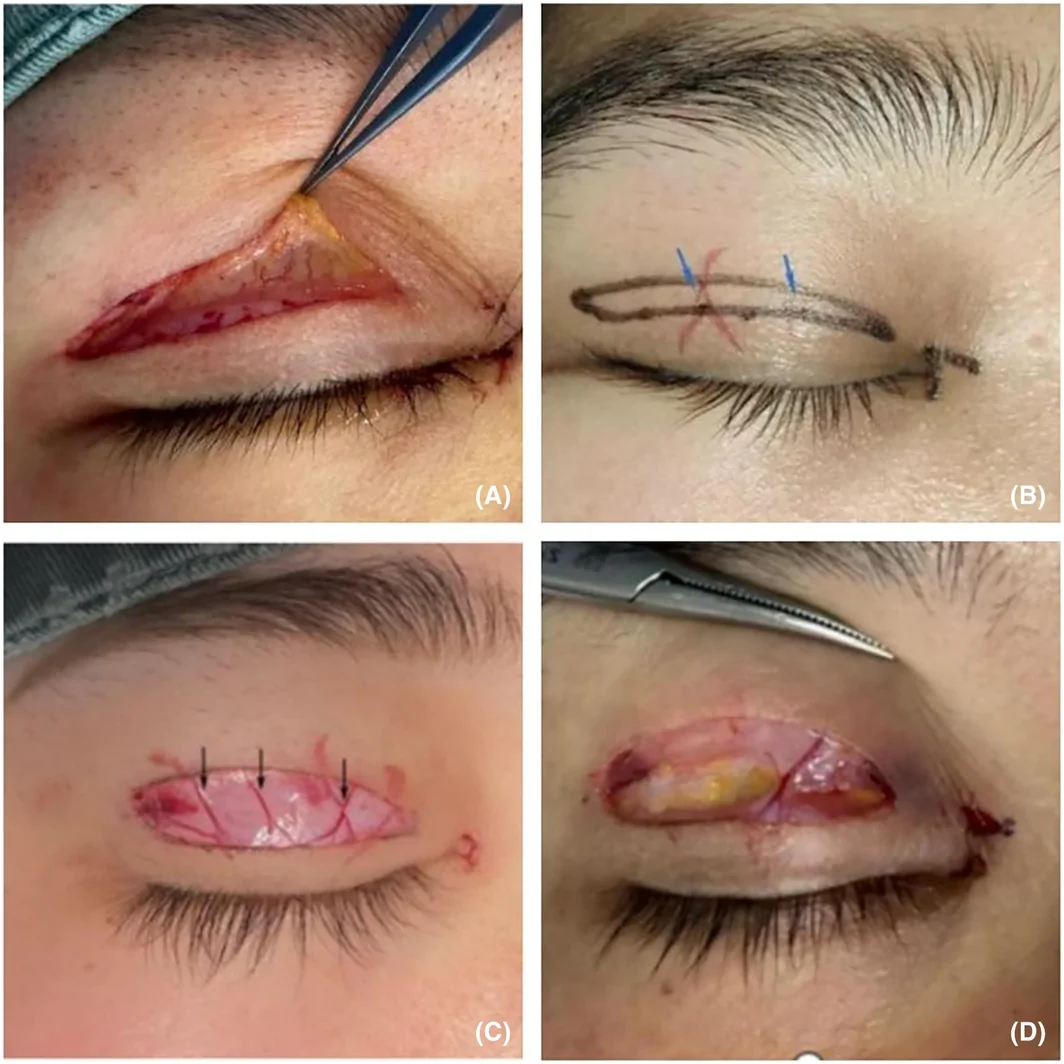
(A) Pre‐tarsus (deep orbicularis oculi) vascular network; (B) Preoperative marking of superficial venous network(Blue arrow); (C) Pre‐orbicularis oculi venous network(Black arrow); (D) Preserved central group of vessels.

#### 2.2.3 Anterior orbicularis oculi vessel preservation

The design methodology remained consistent, and the superficial vein line was delineated on the skin surface during preoperative marking (Figure 1B). Following effective anesthesia, a No. 11 sharp knife was used to make a gentle incision from the skin to the dermis along the predetermined surgical incision, after which the skin was meticulously separated and excised below the dermis using fine scissors while preserving the longitudinally distributed superficial vascular network within the orbicularis oculi muscle's superficial layer (Figure 1C). A cluster of thicker central vessels were carefully selected for preservation, along with a small amount of surrounding fascia tissue. Other adjacent vessels were electrocoagulated for hemostasis purposes (Figure 1D). The spaces on both sides of these vessels were utilized to trim excess orbicularis oculi muscle, intraorbital fat, and part of the ROOF layer fat in order to prevent any vascular damage during surgery. Pre‐tarsus vessels were not intentionally retained. The remaining steps mirrored those employed previously.

### 2.3 Postoperative management

The incision was treated with erythromycin eye ointment and iced for 20 min immediately after the operation, and iced twice a day for 20 min each time for the first 2 days after the operation. The sutures were removed on the 7th day after the operation. No anti‐scar drugs were used in all patients after the operation.

## 3 OBSERVATION INDICATORS

The bleeding volume and duration of surgery for each patient were recorded. The degree of upper eyelid swelling and patient satisfaction were assessed immediately post‐surgery, 1‐day, and 7‐day,1 month, and 6 months after surgery. At the six‐month follow‐up, complications such as postoperative hematoma, infection, scar formation, asymmetry or loss of double eyelid line definition, and local sclerosis were documented.

### 3.1 Swelling

The degree of upper eyelid swelling was evaluated at each time point after the operation.(Table 1).

**TABLE 1:** Swelling degree of upper eyelids rating scale.

Grade	Performance	Score
I	The absence of swelling, bulging, the softness of eyelid tissue, and a close‐to‐normal appearance.	0
II	The upper eyelid exhibits mild edema, without any signs of bruising or skin discoloration. There is no significant periorbital swelling, and the texture appears normal.	1
III	The upper eyelid exhibits moderate swelling, accompanied by mild skin cyanosis and slight edema of the periorbital tissue. However, the skin texture remains discernible, although the periorbital tissue edema has resolved, there is no significant reduction in the width of sub‐incisional swelling compared to its preoperative state.	2
IV	Severe upper eyelid edema, with shiny and visibly cyanotic skin, accompanied by pronounced periorbital tissue swelling and loss of skin texture. Alternatively, compared to previous observations, although the periorbital tissue swelling has decreased or resolved, there is no significant reduction in the sub‐incisional edema width.	3

### 3.2 Satisfaction

The immediate, 1‐day, and 7‐day postoperative scores were assigned based on intraoperative pain, fluency of the double eyelid line, swelling, hematoma presence, and symmetry of the double eyelid line. Swelling during recovery process, postoperative complications, symmetry of the double eyelid shape, and scar appearance were evaluated at 1‐month and 6‐months postoperatively. A five‐point Likert type scale was used, and a score ≥4 was considered satisfactory. The specific scoring criteria are shown in Table 2.

**TABLE 2:** Likert‐type scale.

Degree of satisfaction	Score
Very satisfied	5
Satisfied	4
Generally satisfied	3
Dissatisfied	2
Very dissatisfied	1

### 3.3 Statistical analysis

The statistical analysis was performed using IBM SPSS 27.0 software. Measurement data were presented as mean ± standard deviation (x ± s). One‐way ANOVA was employed to test the three groups of data when they met the assumptions of normality and homogeneity of variances, with a significance level set at *p* < 0.05. Fisher's exact test was used for analyzing count data, with a significance level set at *p* < 0.05.

## 4 RESULTS

### 4.1 Information

Of the 309 patients, 39 were lost to follow‐up. The main reasons were that patients did not want to continue to participate in the follow‐up due to their own reasons, automatic withdrawal from the study, and loss of contact information. Finally, 92 patients remained in group A, 90 in group B, and 88 in group C. There were no significant differences in age distribution and gender composition among the groups (Table 3).

**TABLE 3:** Patient's information.

Group	Number (*n*)	Gender		Age
		Female	Male	
A	92	79	13	26.59 ± 6.32
B	90	80	10	26.51 ± 5.70
C	88	77	11	25.75 ± 4.74

### 4.2 Comparison of operation time and bleeding volume

Statistically, group A exhibited a significantly shorter duration of operation compared to groups B and C; however, it was associated with higher intraoperative bleeding. Further analysis revealed no significant difference in intraoperative bleeding between groups B and C (*t* = 0.34, *p* = 0.72), while group C demonstrated a significantly shorter operation time than group B (*t* = 3.73, *p* < 0.001) (Table 4).

**TABLE 4:** Operation time and bleeding volume.

Group	Time (min)	Bleeding volume (mL)
A	33.47 ± 4.48	8.51 ± 3.36
B	40.21 ± 5.02	7.25 ± 2.35
C	36.82 ± 4.86	7.11 ± 1.94
F value	28.32	5.02
*p* value	<0.001	0.007

### 4.3 Degree of swelling

There was no significant difference in swelling degree among the groups immediately postoperative 1 day and 6 months after surgery, but 7 day, 15 day and 1 month after surgery, the swelling degree of group B and group C was significantly lower than that of group A.(Table 5; Figure 2).

**TABLE 5:** Degree of swelling at different times after surgery.

Group	Postoperative	1 day	7 day	15 day	1 month	6 month
A	1.58 ± 0.56	2.52 ± 0.60	1.18 ± 0.59	1.15 ± 0.57	0.71 ± 0.52	0.30 ± 0.46
B	1.51 ± 0.54	2.67 ± 0.63	1.58 ± 0.56	0.90 ± 0.57	0.48 ± 0.59	0.22 ± 0.41
C	1.57 ± 0.59	2.43 ± 0.59	1.55 ± 0.62	0.95 ± 0.43	0.45 ± 0.56	0.26 ± 0.44
*F* value	0.229	2.551	3.594	3.726	4.002	0.541
*p* value	0.795	0.080	0.029	0.026	0.020	0.583

**FIGURE 2:**
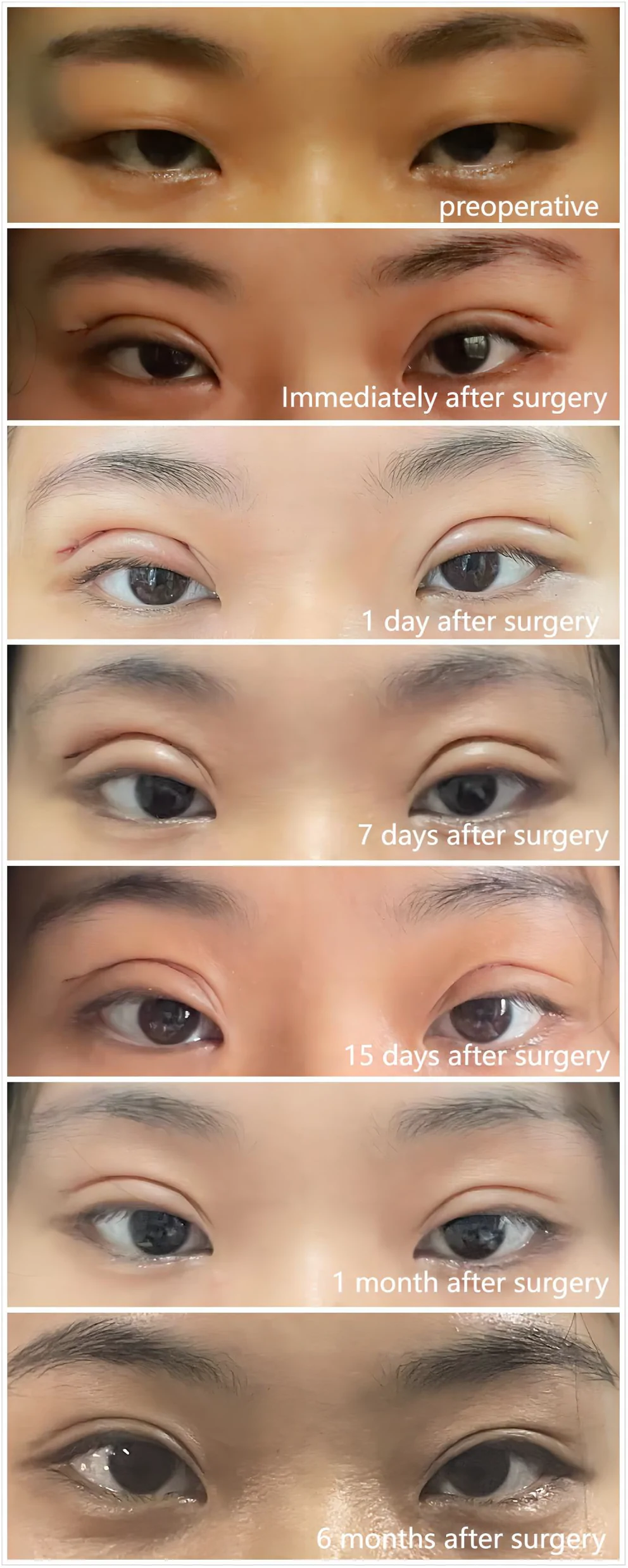
Conventional operation at each time point.

### 4.4 Satisfaction

There were no significant differences in satisfaction levels among the groups immediately after surgery, 1 day and 6 months post‐surgery; however, 7 day and 1 month post‐surgery, groups B and C exhibited significantly higher satisfaction compared to group A (Table 6).

**TABLE 6:** Satisfaction at different times after surgery.

Group	Postoperative	1 day	7 day	1 month	6 month
A	3.53 ± 1.06	3.52 ± 1.03	3.67 ± 1.03	3.39 ± 1.04	4.34 ± 0.84
B	3.75 ± 0.92	3.78 ± 0.93	4.06 ± 0.92	4.16 ± 0.93	4.33 ± 0.81
C	3.82 ± 1.08	3.83 ± 1.09	4.11 ± 1.01	4.23 ± 0.91	4.42 ± 0.76
*F* value	1.249	1.663	3.513	3.556	0.257
*p* value	0.289	0.193	0.032	0.031	0.773

### 4.5 Complications

The incidence of hematoma was highest in group A, while there were no significant differences observed in the incidences of infection, scar, double eyelid line asymmetry, and disappearance among the groups. Furthermore, the incidence of local sclerosis was significantly higher in group C compared to groups A and B (Table 7).

**TABLE 7:** Complications.

Group	Hematoma (*n*, %)	Infection (*n*, %)	Scar (*n*, %)	Asymmetry (*n*, %)	Disappearance (*n*, %)	Local sclerosis (*n*, %)
A (*n* = 92)	6 (6.52%)	0 (0.00%)	5 (5.43%)	6 (6.52%)	5 (5.43%)	1 (1.08%)
B (*n* = 90)	2 (2.22%)	0 (0.00%)	4 (4.44%)	5 (5.55%)	4 (4.44%)	1 (1.11%)
C (*n* = 88)	2 (2.27%)	0 (0.00%)	5 (5.68%)	4 (4.54%)	4 (4.54%)	4 (4.54%)

## 5 DISCUSSION

The palpebral fissure of East Asians exhibits significant racial characteristics:a narrower width, thicker skin and orbicularis oculi muscle, plump orbital septum fat, and rich pre‐orbital fat. These features contribute to the perception of small and bloated eyes.^7^, ^8^, ^9^ However, in popular east asian aesthetics, larger eyes with clearly defined eyelids are considered more attractive.^10^ Blepharoplasty can create a layered appearance for the eyelids that visually enlarges the eyes while also expanding the palpebral fissure and visual field for a more mysterious look.^11^

However, with the advancement of time and the rapid acceleration of life's pace, both immediate and long‐term postoperative effects have become a focal point for doctors and patients alike. Most patients seek to minimize swelling in the surgical area while also aiming for smoother and more natural double eyelid lines.“Three‐point minimally invasive” and “total incision” are the most common surgical methods.^12^, ^13^ Nevertheless, the three‐point method imposes higher requirements on the eye's basic conditions and is only suitable for patients with non‐relaxed upper eyelid skin or low content of orbital septum fat or ROOF layer fat. The effect of total resection is precise, resulting in a well‐defined double eyelid crease and maximum removal of excess fat. However, it also presents drawbacks such as conspicuous scarring at the incision site and significant postoperative swelling.^14^, ^15^

Causes of postoperative swelling: blood exudation; exudation of bodily fluids; impairment of venous and lymphatic drainage pathways, hindered tissue fluid absorption.^16^ By comparing the preservation of both the superficial and deep venous networks of the orbicularis oculi muscle, it was observed that preserving both the deep and superficial blood vessels effectively reduces local swelling in the early postoperative period. This enables patients to quickly resume their normal daily activities and social life, leading to a significant improvement in satisfaction.The superior palpebral vein drainage can be categorized into two levels: the anterior tarsal and the posterior tarsal. The anterior tarsal vein can be further subdivided into two levels: the anterior orbicularis oculi vein and the posterior orbicularis oculi vein. The anterior orbicularis oculi vein is situated in the subcutaneous layer of the upper eyelid, being superficial, while some scholars argue that the posterior orbicularis oculi vein coexists with arteries.^17^ The preservation of either the superficial or deep blood vessels of the orbicularis oculi muscle is observed to provide an anatomical foundation for reducing postoperative swelling. However, there was no significant disparity in the degree of swelling among each group during a long‐term follow‐up, which may be attributed to the sprouting and regeneration of most blood vessels into mature and stable forms within 15 days after surgery. (Figures 3 and 4).

**FIGURE 3:**
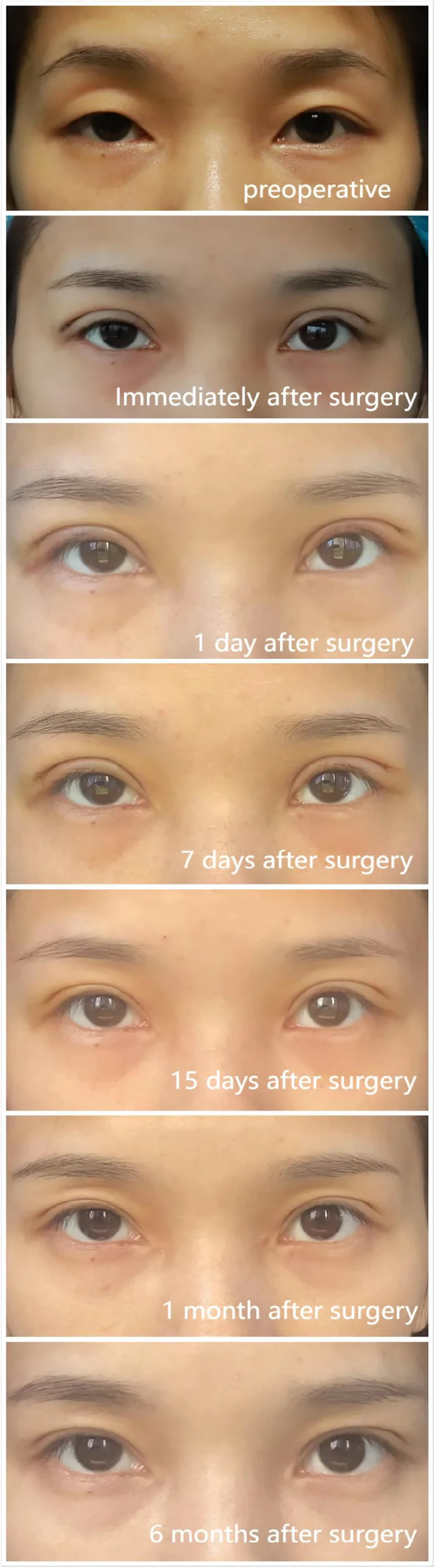
Deep vessel preservation at each time point.

**FIGURE 4:**
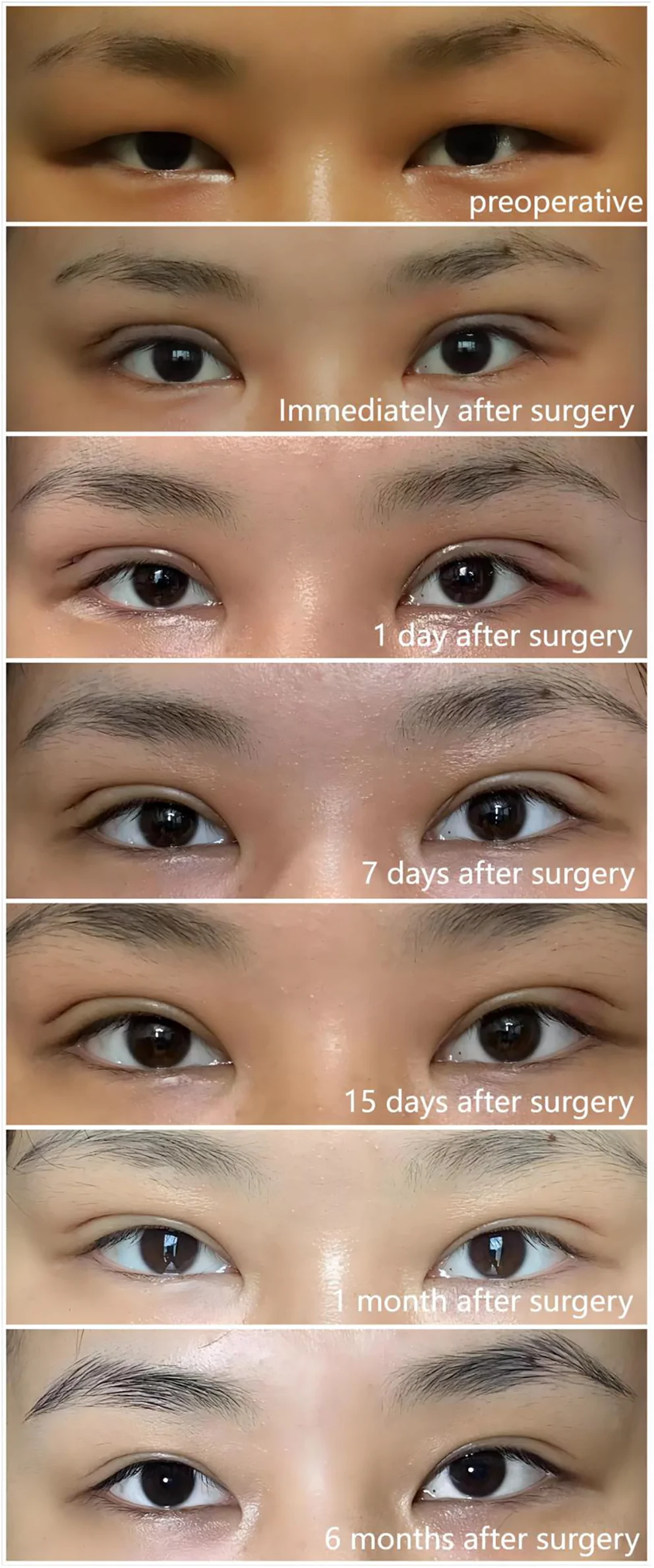
Anterior orbicularis oculi vessel preservation at each time point.

The satisfaction of group B and C were significantly higher than that of group A at both 7 days and 1 month post‐operation. It takes approximately 3 weeks for the lymphatic collecting duct to undergo reconstruction following an injury. The eyelid lymphatic system possesses a substantial blood supply and has the ability to regulate its own fluid filtration and reabsorption capacity, which plays a crucial role in various pathophysiological conditions.^18^, ^19^ Within this fascia tissue, there exist not only venous vessels but also accompanying lymphatic vessels. This fascia tissue provides an anatomical foundation for promoting venous return and lymphatic drainage, thereby exerting a positive effect on reducing local swelling.

Further comparison revealed no significant difference in the degree of postoperative swelling improvement between group B and group C, but the operation time was significantly shorter in group C compared to group B. Jiafu Y^20^ et al employed micromanipulation to preserve 5 to 6 longitudinal blood vessels anterior to the orbicularis oculi muscle, which effectively reduced postoperative edema but required high difficulty and longer operation time. In contrast, only thicker central blood vessels were retained in group C, reducing operative complexity while ensuring sufficient space on both sides of the blood vessels for addressing excess fat tissue in the orbital septum and ROOF. The deep blood vessel's location is deeper with a smaller diameter, necessitating microscopic instrument manipulation when necessary, resulting in prolonged operation time.

The hematoma rate of group A was significantly higher than that of group B and C, which may be related to more vascular destruction during the operation. However, the incidence of local sclerosis under the double eyelid in group C was higher than that in group A and B, which may be related to the amount of perivascular fascial tissue reserved during the operation.The local sclerosis were effectively improved by secondary surgery. However, during the second stage repair, it was observed that the diameter of the original retained blood vessels was significantly larger than that in the initial operation, indicating compensatory thickening of the retained blood vessels after double eyelid plasty.The focus of attention during the operation lies in the extent of perivascular fascial tissue retention. Based on experience, the author has observed that if the amount of perivascular tissue retention exceeds 2 mm, although a mild swelling reaction may be achieved, there is an increased risk of postoperative local sclerosis.

## 6 CONCLUSION

Selective retention of palpebral vein network has a positive effect on reducing postoperative swelling, and there is no significant difference between superficial and deep facial veins in reducing postoperative swelling. However, the operation time of retaining the superficial vessels of the orbicularis oculi muscle is shorter, and the amount of perivascular tissue should be strictly controlled during the operation to avoid the occurrence of local sclerosis.

## AUTHOR CONTRIBUTIONS

Dan LI and Han YAN completed the data collection, analysis and writing of the paper.E YANG and Xinhao LIU completed the data collection and analysis of this paper. Hengshu ZHANG completed the operation of all patients, data collation, and paper review.

## FUNDING INFORMATION

None.

## CONFLICT OF INTEREST STATEMENT

None.

## ETHICS STATEMENT

The authors confirm that the ethical policies of the journal, as noted on the journal's author guidelines page, have been adhered to. This study was obtained approval from the Ethics Committee.

## Data Availability

The data that support the findings of this study are available from the corresponding author upon reasonable request.
